# The role of low subcortical iron, white matter myelin, and oligodendrocytes in schizophrenia: a quantitative susceptibility mapping and diffusion tensor imaging study

**DOI:** 10.1038/s41380-025-03195-7

**Published:** 2025-09-05

**Authors:** Luke J. Vano, Robert A. McCutcheon, Jan Sedlacik, Grazia Rutigliano, Stephen J. Kaar, Valeria Finelli, Maria C. Lobo, Alaine Berry, Ben Statton, Amir Fazlollahi, Ian P. Everall, Oliver D. Howes

**Affiliations:** 1 Department of Psychosis Studies, Institute of Psychiatry, Psychology & Neuroscience, King’s College London, London, UK; 2 Psychiatric Imaging Group, MRC Laboratory of Medical Sciences, Hammersmith Hospital, London, UK; 3 Institute of Clinical Sciences, Faculty of Medicine, Imperial College London, London, UK; 4 South London and Maudsley NHS Foundation Trust, London, UK; 5 Department of Psychiatry, University of Oxford, Oxford, UK; 6 Oxford Health NHS Foundation Trust, Warneford Hospital, Oxford, UK; 7 Mansfield Centre for Innovation - MR Facility, MRC Laboratory of Medical Sciences, Hammersmith Hospital, London, UK; 8 Division of Psychology and Mental Health, Faculty of Biology, Medicine, and Health, University of Manchester, Manchester, UK; 9 Department of Radiology, Royal Melbourne Hospital, University of Melbourne, Parkville, VIC Australia; 10 Queensland Brain Institute, The University of Queensland, Brisbane, QLD Australia

**Keywords:** Neuroscience, Schizophrenia

## Abstract

Iron—the most abundant magnetic brain substance—is essential for many biological processes, including dopamine and myelin synthesis. Quantitative susceptibility mapping (QSM) MRI has recently linked altered subcortical magnetic susceptibility (χ) to schizophrenia. Since χ is increased by iron and decreased by myelin, abnormal levels of either could underlie these QSM differences. In white matter tracts, magnetic susceptibility anisotropy (δχ) serves as a myelin-specific marker that is insensitive to iron content. To clarify the origin of case-control χ differences, we employed QSM in 85 individuals with schizophrenia, from first-episode mental health teams, and 86 healthy controls. A subset also underwent diffusion tensor imaging (DTI) to calculate subcortical tissue mean diffusivity, which inversely correlates with myelin concentration and fractional anisotropy. White matter δχ was calculated by combining QSM and DTI. Schizophrenia was associated with lower subcortical χ (d = −0.36, p = 0.023). This was significant in the caudate nucleus (d = −0.37, p = 0.037), putamen (d = −0.36, p = 0.037), globus pallidus (d = −0.57, p = 0.001), and SN-VTA (as previously reported). Additionally, schizophrenia was linked to higher subcortical mean diffusivity (d = 0.44, p = 0.018), and lower white matter δχ (d = −0.37, p = 0.047). These findings suggest that both subcortical iron and brain myelin levels are lower in schizophrenia. By comparing our voxelwise χ maps with postmortem gene expression data, we reveal that regions with lower subcortical χ in schizophrenia are enriched for oligodendrocyte-related genes (p < 0.001). As oligodendrocytes are both the most iron-rich brain cells and essential for myelin synthesis, our results implicate oligodendrocyte dysfunction in schizophrenia pathophysiology.

## Introduction

Schizophrenia is a mental illness characterized by positive psychotic (e.g., persistent delusions, hallucinations, and disorganized thinking), negative (e.g., social withdrawal or lack of motivation), and cognitive symptoms [1]. Altered dopamine [2] and myelin synthesis [3], via iron-mediated pathways [4], have been implicated in disease pathophysiology. Iron abnormalities are also present, with increased blood iron deficiency seen in people with schizophrenia [5] and pregnant women whose children later develop the condition [6].

Direct investigation of central nervous system iron levels in schizophrenia has produced inconclusive results. Early postmortem [7–9] and CSF [10, 11] studies found no case-control differences, while a recent large study identified increased cortical iron in schizophrenia [12]. Indirect assessment of subcortical iron has also been conducted using in vivo MRI [13–17]. Iron’s paramagnetic properties increase tissue magnetic susceptibility (χ), which can be calculated by quantitative susceptibility mapping (QSM). Additionally, iron induces local magnetic field inhomogeneities within the tissue microstructure, accelerating the decay of transverse magnetization and leading to a higher effective transverse relaxation rate (R2*).

Case-control studies employing iron-sensitive MRI of the subcortical structures have yielded conflicting results, with schizophrenia being linked to lower (R2* [14, 17]; χ [13, 14]) and higher iron-sensitive signal (R2* [16]; χ [15]). This inconsistency may be due to disease-related variations in myelin. Myelin is the principal diamagnetic material in the brain [18], which reduces tissue χ and also causes local magnetic field inhomogeneities within the tissue microstructure, which increases R2*. Combining iron-sensitive MRI with a marker of myelin not influenced by iron, such as mean diffusivity derived from diffusion tensor imaging (DTI), could clarify the origins of these differences. Mean diffusivity measures the average water diffusion in all directions within a voxel and inversely correlates with myelin levels and overall tissue density [19].

Analyzing the myelin-rich but comparatively iron-poor white matter tracts [20, 21] intersecting subcortical structures could further reveal whether myelin abnormalities are linked to schizophrenia. While tissue orientation does not affect the χ of iron, χ from myelin in white matter is influenced by fiber orientation, becoming more negative as fiber alignment becomes more perpendicular to the background magnetic field [22–24]. In white matter, combining voxel χ with fiber orientation, assessed with DTI, enables the calculation of magnetic susceptibility anisotropy (δχ). This metric correlates with myelin levels and is unaffected by iron [25].

Our previous case-control study using the same sample demonstrated that neuromelanin-sensitive MRI (NM-MRI) signal, which reflects neuromelanin-bound iron, was elevated in the substantia nigra and ventral tegmental area (SN-VTA) in schizophrenia [26]. As neuromelanin synthesis is a byproduct of dopamine metabolism [4], and NM-MRI correlated with in vivo dopamine synthesis capacity in our previous study, this suggests that dopamine neurons contain more neuromelanin-bound iron in schizophrenia. Our follow-up analysis revealed that SN-VTA χ was lower in schizophrenia, without significantly different mean diffusivity, and inversely correlated with dopamine synthesis capacity, indicating that SN-VTA non-neuromelanin-bound iron is lower in schizophrenia [27]. The extent and biological basis of χ difference in other subcortical regions in schizophrenia remain unclear.

**Fig. 1:**
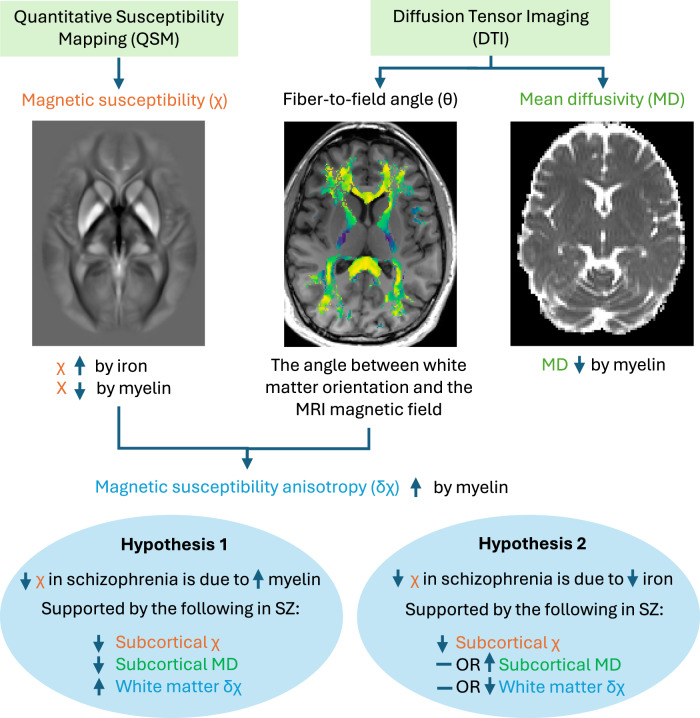
Overview of methods testing the two competing hypotheses. Quantitative Susceptibility Mapping (QSM) was used to derive magnetic susceptibility (χ), which increases with tissue iron and decreases with myelin. Diffusion Tensor Imaging (DTI) was used to calculate mean diffusivity, which inversely correlates with myelin, and the fiber-to-field angle (θ), representing the angle between predominant white matter fiber orientation and the background MRI magnetic field. Combining χ and θ enabled the calculation of magnetic susceptibility anisotropy (δχ), a marker correlated with white matter myelin. QSM and DTI were applied to healthy controls and patients to test whether low χ in schizophrenia results from high myelin (hypothesis 1) or low iron (hypothesis 2). Hypothesis 1 would be supported if patients with schizophrenia demonstrated lower subcortical χ, lower subcortical mean diffusivity, and higher white matter δχ than controls. In contrast, hypothesis 2 would be supported if patients exhibited lower subcortical χ along with either unchanged or higher subcortical mean diffusivity and either unchanged or lower white matter δχ compared to controls.

In this study, we utilized QSM and DTI to investigate whether subcortical χ differences in schizophrenia are driven by altered iron or myelin levels. As outlined in Fig. 1, we compared subcortical χ, subcortical mean diffusivity, and white matter δχ to test two competing hypotheses: (1) lower subcortical χ in schizophrenia results from higher myelin, supported by lower subcortical mean diffusivity and higher white matter δχ in schizophrenia; or (2) lower subcortical χ reflects lower iron, supported by unchanged or higher subcortical mean diffusivity and unchanged or lower white matter δχ in schizophrenia. To link disease-related χ differences to specific gene pathways and cell types, we combined our subcortical χ t-score map with gene expression data from the Allen Human Brain Atlas [28]—a genome-wide map of over 3500 samples taken from postmortem human brains.

## Methods

### Participants

For our cross-sectional case-control study, participants were recruited between August 2019 and May 2023. Patients with a diagnosis of schizophrenia were identified from community mental health teams in London, England, and demographically matched healthy controls were enrolled via advertisements. Eligible participants were aged 18–45 years, with the upper limit chosen to focus on individuals early in the illness course, given potential progressive brain changes in schizophrenia [29]. Female participants could not be breastfeeding or have a positive urine pregnancy test before scanning.

In the patient sample, a study psychiatrist used The Structured Clinical Interview for Diagnostic and Statistical Manual of Mental Disorders-5 (SCID-5) [30] and reviewed clinical notes to confirm a schizophrenia diagnosis and exclude those with a history of any other clinically significant psychiatric disorder. Patients could not have a history of clozapine use, given that dopaminergic alterations differ in patients who do not benefit from first-line antipsychotics [31]. Healthy controls were excluded if they had a history of any diagnosed mental illness or any family history of a psychotic disorder. Exclusion criteria for both groups included neurological, endocrine, or oncological conditions. Participants underwent a urine drug screen (UDS) immediately before the MRI, and anyone testing positive for substances other than delta-9-tetrahydrocannabinol (THC), which can remain detectable for an extended period [32], was excluded. Volunteers were characterized as being a non-smoker, past smoker, or current smoker based on if/when they last smoked at least 5 cigarettes per week for a duration of 3 months [33].

### Clinical measures

We collected demographic data for all participants. Patients with schizophrenia underwent the SCID-5, Positive and Negative Syndrome Scale (PANSS) [34], Brief Negative Symptoms Scale (BNSS) [35], and Clinical Global Impression- Severity scale (CGI-S) [36]. Antipsychotic medication doses were converted to daily chlorpromazine equivalent doses via the method outlined by Leucht et al. [37].

### Image acquisition and processing

Participants were scanned using a 3 T MRI scanner (Magnetom Prisma, Siemens Medical Systems, Germany) with a 64-channel receive head coil. The MRI acquisition protocols and full processing pipelines to generate the QSM (voxel size = 1 × 1 × 1 mm^3^) and DTI images (voxel size = 1.7 × 1.7 × 4 mm^3^) are provided in the Supplementary Methods. χ is reported in parts per billion (ppb) and mean diffusivity in 10^5^ mm^2^/s.

Briefly, each participant’s 3D gradient recall echo (GRE) first echo magnitude image was linearly co-registered to their T1-weighted image. These T1-weighted images were then normalized to the Montreal Neurological Imaging (MNI) space (voxel size = 1 × 1 × 1 mm^3^) [38]. The relevant affine transformation-matrices and warp-fields were applied to each subject’s QSM image to move these into the MNI space where they were smoothed with a 1-mm sigma Gaussian kernel. A white matter mask was generated for each subject’s T1-weighted image using Freesurfer [39] and moved into their QSM space. Investigators (LJV and JS) used best practice guidelines to quality control the QSM image [40]. We employed an SN-VTA mask (Supplementary Fig. S1; available from: https://github.com/lukevano/KCL_Neuromelanin-MRI) generated using neuromelanin-sensitive MRI data from these participants, as detailed in our previous study [26].

The following regions of interest (ROI) from the CerebrA [41] brain atlas were combined with our SN-VTA mask to form the complete subcortical atlas: caudate nucleus, putamen, nucleus accumbens, globus pallidus, and thalamus. This atlas was moved into each subject’s QSM and DTI space for ROI analysis. A striatal functional subregion atlas (limbic, associative, and sensorimotor) [42] was similarly processed.

DTI data were pre-processed using the FMRIB Software Laboratory (FSL) diffusion and tract-based spatial statistics (TBSS) toolboxes [43] to generate mean diffusivity and fractional anisotropy images. Fractional anisotropy measures the directionality of water diffusion, where a value of 0 indicates isotropic diffusion (no preferred direction) and a value of 1 represents maximal anisotropic diffusion (all diffusion occurs along a single direction, such as within a white matter tract). Our calculation of δχ followed the pipeline established by Sibgatulin et al. [44, 45]. Constrained spherical deconvolution modelled the orientation of white matter fibers within a voxel [46]. The first peak of the orientation distribution function determined the angle between the predominant white matter fiber orientation and the MRI main magnetic field, termed the fiber-to-field angle (θ) [46]. The mean diffusivity image was rigidly co-registered to the GRE first echo magnitude image. The fractional anisotropy and θ maps were then transformed into the QSM space by applying the generated affine transformation-matrix.

To ensure analysis was restricted to white matter, white matter masks in QSM space were eroded by a 2-mm radius, and voxels with crossing fibers (defined where fractional anisotropy <0.6), or extreme χ values (beyond the 1st and 99th percentiles: −110 to 30 ppb) were excluded. TractSeg [47] identified specific white matter tracts with >90% labeling probability. These masks were further eroded using a 6-connected structural element. The corpus callosum was subdivided into the genu, body, and splenium, while the anterior limb of the internal capsule was separated from the anterior thalamic radiation, as detailed in our Supplementary Methods. Additional tracts analyzed included the corticospinal tract, superior longitudinal fascicles, cingulum, and optic radiation. Processed masks for a control participant are shown in Supplementary Fig. S2.

The relationship between magnetic susceptibility (χ), magnetic susceptibility anisotropy (δχ), fiber-to-field angle (θ), and orientation-independent χ (χ_iso_) is defined by the equation:

$$\chi =\delta \chi \;{\cos }^{2}\theta +{\chi }_{{{\rm{iso}}}}$$

We used this equation to estimate δχ and χ_iso_ from χ and θ following the steps outlined by Sibgatulin et al. [44, 45]. Specifically, we separated the voxels within each tract into 10 equally populated bins based on their θ values, such that the first bin contained the lowest 10% of θ values, and each subsequent bin represented voxels within the next 10th percentile. Mean θ and χ values were calculated for each bin, and least squares regression was performed with these values to model χ as a function of θ. In this approach, δχ was the slope (first-order coefficient), and χiso was the intercept.

White matter χ is theoretically expected to increase with decreased θ [22–24], resulting in a positive δχ. However, negative δχ has been reported in specific white matter tracts [25, 44, 45], potentially due to errors in χ estimation for tracts running past the highly paramagnetic subcortical structures [25]. Therefore, we first calculated tract δχ using the methods outlined above applied across all voxels from the healthy controls for each tract. Only tracts with a significantly positive δχ coefficient (p < 0.05) were selected for further case-control analysis. For each participant, we averaged δχ values across these selected tracts to obtain the participant white matter δχ.

### Gene expression data

Gene expression data were sourced from the Allen Human Brain Atlas [28], a publicly available dataset profiling the expression of 15,633 genes across over 3500 samples from 6 postmortem, non-diseased adult brains. Following prior analyses [48, 49], we focused on the left hemisphere due to limited right hemisphere sampling. Data were processed as follows using the “abagen” (version 0.1.3) python toolbox [50] as outlined in our previous paper [48]: obtain MNI coordinates for each sample, reannotate probe-to-gene mappings with data from Arnatkevic̆iūtė et al. [51], remove probes not exceeding background noise in 50% of all tissue samples (using intensity-based filtering), and select probes with highest differential stability across donors if multiple identified for the same gene.

From this pipeline, we identified 201 samples inside the left-sided subcortical mask. Each sample was assigned a χ t-score by matching the MNI coordinates of the tissue sample to the corresponding χ t-score voxel MNI coordinates. A robust sigmoid function normalized sample expression values across genes, brain regions, and donors.

### Statistical analysis

The power calculation for our study is detailed in the Supplemental Materials. All statistical tests were conducted as two-sided unless otherwise specified.

#### Baseline clinicodemographic data

Case-control differences were assessed using independent sample t-tests for continuous variables and chi-squared tests for categorical variables.

#### Case-control neuroimaging analysis

##### Primary analysis

Independent sample t-tests and Cohen’s d assessed the significance and effect size of case-control differences in subcortical χ, subcortical mean diffusivity, and white matter δχ.

##### Exploratory analysis

Case-control differences in χ, R2*, mean diffusivity, and fractional anisotropy were examined in each subcortical ROI. Pearson’s r correlated subcortical χ with clinical measures in schizophrenia. Additionally, case-control differences in white matter mean diffusivity and fractional anisotropy were calculated. For these analyses, a Benjamini-Hochberg false discovery rate (FDR) correction was applied (p < 0.05). Robust linear regression evaluated the impact of clinical confounders (age, sex, current or past smoking history, and THC-positive UDS) on subcortical χ in patients.

##### Final model

A robust linear regression modeled the relationship between subcortical χ, case-control status, subcortical mean diffusivity, white matter δχ, and clinical confounders. Missing mean diffusivity and δχ values were imputed using group means.

##### Voxelwise analysis

Robust linear regression predicted subcortical voxel χ based on case-control status, adjusting for clinical confounders. Data outside the 1st and 99th percentiles (−74 and 171 ppb) were censored. Threshold-free cluster enhancement (TFCE) with 10,000 permutations identified significant clusters (FDR-corrected p < 0.05) [52].

#### Gene expression association with χ t-score map

##### Partial least squares regression (PLSR)

PLSR, chosen because it is well-suited to high-dimensional data with multicollinearity [53], linked subcortical voxel case-control χ t-scores with gene expression profiles. Utilizing the NIPALS algorithm in scikit-learn [54], PLSR created composite variables (components) maximizing covariance between χ t-scores for the 201 voxels matched to postmortem samples and expression of 15,633 genes for each sample.

The optimal number of components was determined by averaging Pearson’s r values across five sets of five-fold cross-validation and selecting the number with the highest r. To ensure unbiased results, we averaged Pearson’s r across components 2 to 10 for the ‘true’ model accuracy and compared it against 10,000 random permutations of t-scores and 10,000 spatially autocorrelated t-score maps generated by the BrainSMASH toolbox [55].

##### Gene enrichment analysis

To quantify the association between gene expression and χ t-scores, we calculated a z-score for each gene by dividing its variable importance in projection (VIP) score by its standard error, derived from 10,000 bootstrapped runs (resampling χ t-scores with replacement). Genes were then ranked by z-scores, where genes with high z-scores are associated with high χ t-scores, in descending and ascending lists.

Gene Ontology (GO) enrichment analysis identified cellular locations, molecular functions, and biological processes associated with the gene sets. Genes not expressed in the brain on the Human Protein Atlas RNA GTEx database (www.proteinatlas.org/download/rna_brain_gtex.tsv.zip) were removed. These updated lists were inputted into GOrilla to generate enriched GO terms [56]. Categories with FDR-corrected p < 0.05, dispensability <0.3, and over three overlapping genes were clustered using SimREI in REViGO [57], to yield non-redundant GO terms.

##### Cell-type analysis

To explore cell-specific contributions to χ differences in schizophrenia, we investigated if cell-specific genes were associated with higher or lower z-scores. Lists for inhibitory and excitatory neurons were obtained from Lake et al. [58], while lists for astrocytes, endocytes, microglia, and oligodendrocytes were from Damanis et al. [59]. We calculated how far each cell-specific gene list’s median rank deviated from the z-score ranked gene list center. Negative deviation linked the cell type to higher χ in schizophrenia, while positive deviation connected it to lower χ. Deviations were compared to those from 10,000 randomly selected gene sets of the same size to assess significance.

## Results

### Participant details

A total of 171 participants (control n = 86; schizophrenia n = 85) underwent QSM. As the DTI protocol was added later, only 116 participants had DTI (control n = 43; schizophrenia n = 73). Participant demographic details and clinical assessments are included in Table 1. There were no significant cohort differences for sex, age, or ethnicity.

**Table 1:** Demographic, clinical, and experimental measures for the study participants.

	Healthy Controls (n = 86)	Schizophrenia (n = 85)	Test Statistic
Male	60 (70%)	61 (72%)	χ^2^_1_ = 0; p = 0.91
Age	32.1 (6.4)	31.5 (6.9)	t = −0.63; p = 0.53
**Ethnicity**			
Asian	11 (13%)	11 (13%)	χ^2^_3_ = 0.18; p = 0.98
Black	42 (49%)	43 (50%)	
White	28 (32%)	27 (32%)	
Mixed	5 (6%)	4 (5%)	
**Neuroimaging measures**			
n with QSM	86	85	
n of QSM passing QC	80	79	
n with DTI	43	73	
n of DTI passing QC	43	73	
**Clinical measures**			
PANSS Total	Not performed for Healthy Controls	73.5 (14.1) [36-100]	
PANSS Positive		17.4 (5.6) [7-31]	
PANSS Negative		20.3 (5.6) [8-38]	
PANSS General		36.0 (7.1) [16-49]	
BNSS		30.9 (14.5) [1-65]	
CGI-S		4.0 (0.9) [2-6]	
Duration of illness (years)		2.7 (2.4) [0.2-17]	
With over 5 years of illness and passing QSM QC		14 (18%)	
**Current medication**			
Aripiprazole	None	23	
Olanzapine		21	
Risperidone		8	
Paliperidone		7	
Lurasidone		3	
Flupentixol		2	
Zuclopenthixol		1	
Unmedicated		20	
Chlorpromazine daily equivalent doses (mg)		302.1 (145.3) [0-675]	

Values are expressed as number (and %) or mean (with standard deviation in brackets) [and range in square brackets], besides duration of illness which was expressed as median (and interquartile range). Abbreviations: *n* number of participants, *QSM* quantitative susceptibility mapping, *QC* quality control, *DTI* diffusion tensor imaging, *PANSS* positive and negative syndrome scale, *BNSS* brief negative symptoms scale, *CGI-S* clinical global impression- severity scale, *χ* chi-squared test, *t* independent sample t-test.

### Case-control differences for neuroimaging measures

Table 2 details the case-control differences in subcortical χ and mean diffusivity. Patients with schizophrenia had lower subcortical χ (d = −0.36, t = −2.29, p = 0.023) and higher subcortical mean diffusivity (d = 0.44, t = 2.41, p = 0.018) compared to controls. Our exploratory subregion χ analysis (Supplementary Fig. S3) associated schizophrenia with significantly lower χ in the caudate nucleus (d = −0.37, t = −2.35, p = 0.037), putamen (d = −0.36, t = −2.27, p = 0.037), globus pallidus (d = −0.57, t = −3.6, p = 0.001), and, as we previously reported [27], the SN-VTA (d = −0.66, t = −4.18, p < 0.001). No significant χ differences were found in the thalamus (p = 0.388) or nucleus accumbens (p = 0.388). Our analysis of striatal functional subregions linked schizophrenia to lower χ in the associative subregion (d = −0.42, t = −2.62, p = 0.029) but no difference in the sensorimotor (p = 0.091) or limbic subregions (p = 0.958; Supplementary Table S1). Higher thalamic mean diffusivity (t = 2.64, uncorrected p = 0.009) lost significance after FDR correction (p = 0.054), with no significant mean diffusivity differences in other ROIs (Supplementary Fig. S4).

**Table 2:** Comparison of group mean regional χ, calculated from quantitative susceptibility mapping (QSM) MRI, and mean diffusivity, generated from diffusion tensor imaging (DTI).

Subcortical Magnetic Susceptibility (χ; ppb) and Mean Diffusivity (MD; 10^5 ^mm^2^/s)								
		Controls		Patients		Test Statistics		
		Mean	SD	Mean	SD	d	t-test	p-value
Whole Subcortex	χ	28.83	4.73	27	5.29	−0.36	−2.29	0.023
	MD	79.5	6.04	83.05	10.23	0.44	2.41	0.018
Thalamus	χ	−2.25	6.35	−1.26	6.36	0.16	0.98	0.388
	MD	80.54	5.81	83.36	7.73	0.49	2.64	0.054
Caudate Nucleus	χ	38.29	7.49	35.50	7.51	−0.37	−2.35	0.037
	MD	76.92	4.15	77.87	3.85	0.26	1.35	0.546
Putamen	χ	32.15	8.77	29.19	7.58	−0.36	−2.27	0.037
	MD	72.42	1.42	72.38	1.36	0.01	0.07	0.947
Nucleus Accumbens	χ	−2.95	13.08	−1.20	12.32	0.14	0.87	0.388
	MD	76.29	2.57	76.11	2.19	−0.17	−0.89	0.754
Globus Pallidus	χ	123.32	16.53	113.18	18.92	−0.57	−3.6	0.001
	MD	76	2.41	75.61	2.19	−0.03	−0.13	0.947
SN-VTA	χ	114.23	23.18	99.53	21.19	−0.66	−4.18	<0.001
	MD	78.08	3.63	78.14	3.01	0.07	0.37	0.947

P-values for the subcortical subregions corrected for multiple comparisons using the Benjamini-Hochberg method. Abbreviations: *ppb* parts per billion, *SN-VTA* substantia nigra and ventral tegmental area.

**Fig. 2:**
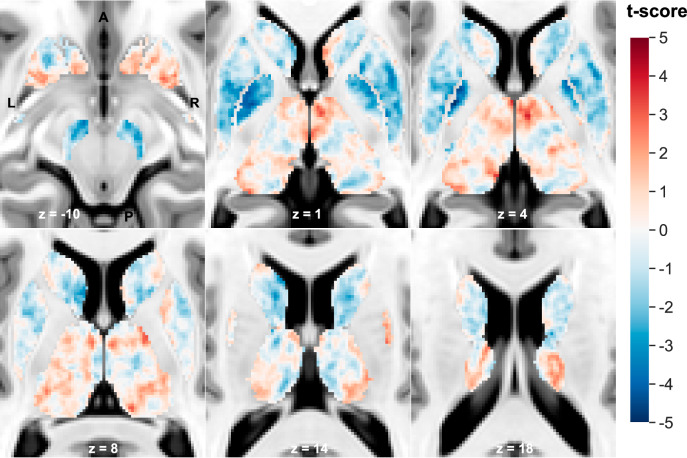
Subcortical magnetic susceptibility (χ) t-score map on the T1-weighted Montreal Neurological Imaging (MNI) template at the annotated slice level and orientation. Voxels where schizophrenia was associated with higher χ relative to controls are in red, with a peak in the right anterior thalamus (peak voxel x = 5, y = −8, z = −2; t = 3.92). Voxels linked to lower χ are in blue, with a peak in the left globus pallidus (peak voxel x = −21, y = −3, z = 4; t = −4.98).

Figure 2 displays the χ t-score map. High-magnitude negative t-scores are seen predominantly in the caudate nucleus, putamen, globus pallidus, and SN-VTA, while positive t-scores were mainly in the anterior thalamus and nucleus accumbens. Supplementary Fig. S5 and Supplementary Table S2 show the results from our exploratory cluster analysis. Seven clusters with lower χ in schizophrenia were identified bilaterally in the SN-VTA, putamen, globus pallidus, and the left caudate nucleus, while two clusters with higher χ were found in the right anterior thalamus and right nucleus accumbens.

Our robust linear regression model in the schizophrenia group showed that duration of illness did not significantly affect subcortical χ (p = 0.327; Supplementary Table S3). χ did not correlate with clinical measures for any ROI (Supplementary Fig. S6). R2* was only significantly different in the globus pallidus, being lower in patients than controls (d = −0.39, t = −2.45, p = 0.016; Supplementary Table S4). A trend toward lower R2* in the patient group was also observed for the whole subcortex (d = −0.28, t = −1.78, p = 0.077). Case-control differences in subcortical fractional anisotropy are reported in Supplementary Table S5 and Fig. S7.

The distribution of fiber-to-field angles (θ) in each white matter ROI for the controls (Supplementary Fig. S8) appeared similar to those from Sibgatulin et al. [45]. As hypothesized, χ was more negative as θ increased (Supplementary Fig. S9) and δχ was positive (Supplementary Table S6) for controls in the following tracts: splenium of the corpus callosum (δχ = 13.35, p = 0.014), superior longitudinal fascicle (δχ = 9.93, p < 0.001), cingulum (δχ = 26.36, p < 0.001), and optic radiation (δχ = 32.14, p < 0.001). Therefore, the average δχ across these tracts was used for the case-control analysis. A negative δχ was observed in the corticospinal tract (δχ = −25.77, p = 0.014), anterior limb of the internal capsule (δχ = −36.96, p = 0.014), and genu of the corpus callosum (δχ = −54.78, p = 0.001). A significant δχ was not identified in the anterior thalamic radiation (p = 0.31) or body of the corpus callosum (p = 0.659).

We found that δχ was significantly lower in patients with schizophrenia than controls (d = −0.37, t = −2.01, p = 0.047; Supplementary Table S7). We did not observe a group difference in white matter χ (p = 0.419) or χiso (p = 0.419). In our white matter DTI analysis (Supplementary Table S8), fractional anisotropy was lower in schizophrenia relative to controls (d = −0.45, t = −2.41, p = 0.036) while no group difference was observed in mean diffusivity (d = 0.19, t = 0.99, p = 0.325).

Subcortical χ remained significantly lower in schizophrenia (t = −2.14, p = 0.032; Table 3) when controlling for potential clinical confounders, subcortical mean diffusivity, and white matter δχ in our robust linear regression model. A positive relationship was identified between subcortical χ and age (t = 2.45, p = 0.014); see Supplementary Fig. S10 (r = 0.21, p = 0.008).

**Table 3:** Results from the robust linear model built to predict subcortical magnetic susceptibility (χ) with case-control status, potential confounders, subcortical mean diffusivity, and white matter magnetic susceptibility anisotropy (δχ; controls n = 80; schizophrenia n = 79).

Variable	Coefficient	Standard Error	t-score	p-value	[95% CI]	
(Intercept)	32.09	10.08	3.18	0.001	12.34	51.85
Schizophrenia Group Status	−1.64	0.76	−2.14	0.032	−3.13	−0.14
Current Smoker	0.52	0.96	0.54	0.590	−1.36	2.39
Past Smoker	0.83	1.11	0.75	0.452	−1.34	3
THC-positive UDS	0.79	0.93	0.85	0.393	−1.02	2.6
Male Sex	0.65	0.8	0.81	0.415	−0.92	2.22
Age	0.13	0.05	2.47	0.014	0.03	0.24
Subcortical Mean Diffusivity	−0.12	0.13	−0.91	0.364	−0.37	0.14
White Matter δχ	23.28	29.09	0.8	0.423	−33.73	80.3

### Subcortical gene expression predicts χ difference in schizophrenia

**Fig. 3:**
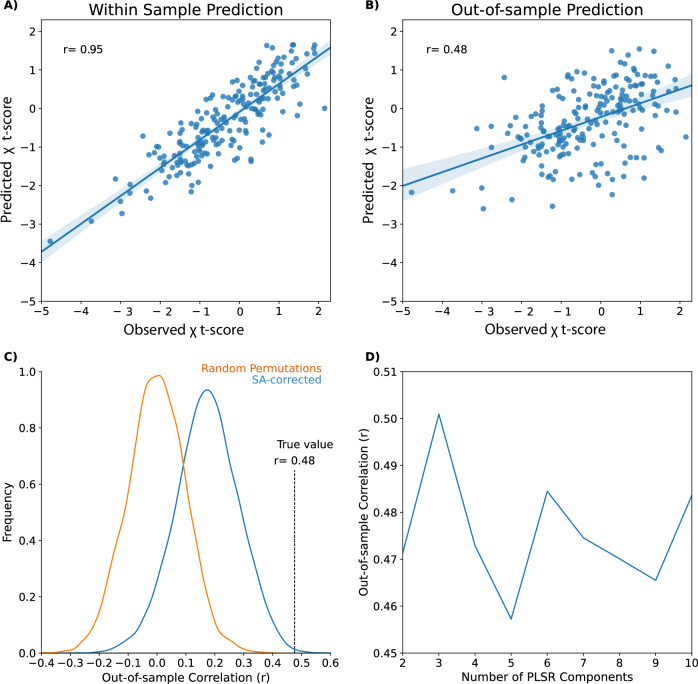
Plots highlighting the ability of partial least squares regression (PLSR) models to predict subcortical magnetic susceptibility (χ) t-score based on subcortical tissue gene expression. Sample χ t-score predictions averaged across PLSR models made with 2–10 components by a fivefold cross-validated approach against the **A** within sample and **B** out-of-sample observed values. **C** The out-of-sample Pearson’s r correlation between true and predicted χ t-scores (r = 0.48; vertical dashed line) was statistically significant when compared to null models generated by 10,000 random permutations of the subcortical voxels (orange histogram; p < 0.001) and when spatial autocorrelation (SA) was preserved (blue histogram; p = 0.001). **D** Out-of-sample prediction accuracy was greatest when the PLSR model was built with three components, so this hyperparameter was used for our endpoint analysis.

We identified 201 samples within the left subcortical mask, each with normalized expression data for 15,633 genes. The correlation between out-of-sample true and predicted t-scores from the PLSR model (r = 0.48; Fig. 3B) was statistically significant when tested against both 10,000 random (p < 0.001; Fig. 3C) and spatial autocorrelation-preserved permutations of χ t-scores (p = 0.001; Fig. 3C). Three components for the PLSR model had the greatest out-of-sample predictive accuracy (Fig. 3D) and were thus used for the final predictive model.

**Fig. 4:**
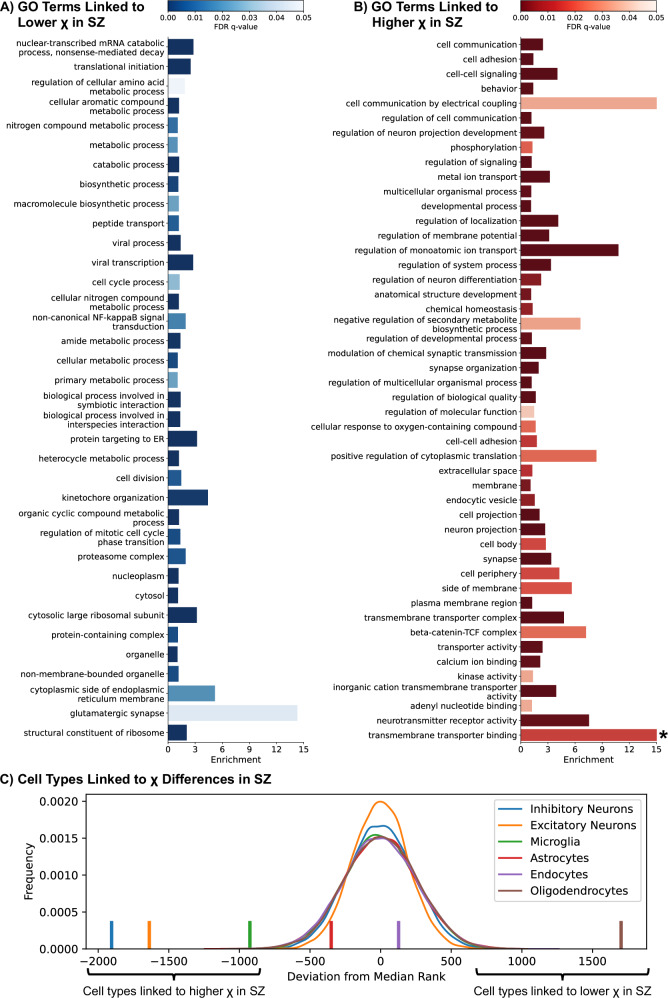
Gene ontology enrichment and cell-type associations with regional magnetic susceptibility (χ) differences in schizophrenia. Enriched Gene Ontology (GO) terms associated with regions of **A** lower χ and **B** higher χ in schizophrenia relative to controls. Only terms with FDR-corrected P < 0.05, REViGO dispensability <0.3, and with four or more overlapping genes are shown. **C** Graph showing how far the median rank of each cell-specific gene list deviated from the center of the z-score ranked gene list (vertical lines). The frequency polygons display the null distribution of deviations generated from 10,000 randomly selected gene sets of the same length of genes as each cell list. Regions of lower χ in schizophrenia were associated with oligodendrocytes (deviation = 1703, p < 0.001) while higher χ was associated with inhibitory neurons (deviation = −1906, p < 0.001), excitatory neurons (deviation = −1639, p < 0.001), and microglia (deviation = −926, p < 0.001). χ differences were not linked to endocytes (deviation = 128, p = 0.627) or astrocytes (deviation = −351, p = 0.177). *, graph truncated to an enrichment of 15, despite the GO term transmembrane transporter binding having an enrichment of 32.67, to improve visualization.

Regions with lower χ in schizophrenia showed the highest enrichment for the GO term glutamatergic synapse (enrichment=14.3, p = 0.043; Fig. 4A) and the oligodendrocyte-related genes (p < 0.001; Fig. 4C). Regions showing higher χ had the greatest enrichment for the transmembrane transporter binding (enrichment = 32.67, p = 0.016; Fig. 4B). These regions were also enriched for genes related to inhibitory neurons, excitatory neurons, and microglia (p < 0.001 for all; Fig. 4C). The following six GO terms in this list (Fig. 4B) were also identified as being linked to schizophrenia by the largest genome-wide association study (GWAS) to date (Supplementary Table S9; [60]): regulation of localization, regulation of neuron differentiation, modulation of chemical synaptic transmission, neuron projection, synapse, transmembrane transporter complex.

## Discussion

Using QSM, we found that subcortical χ is lower in schizophrenia compared to controls, which could be explained by higher myelin levels (hypothesis 1) or lower iron levels (hypothesis 2) in the condition. Our DTI analysis revealed higher subcortical mean diffusivity, which inversely correlates with myelin concentration, and lower white matter δχ, a direct marker of myelin levels, in schizophrenia. These findings suggest that myelin is lower in schizophrenia, which would increase χ. Thus, the lower subcortical χ observed in schizophrenia is likely driven by low iron and partially masked by concurrent low myelin levels.

By comparing case-control χ differences with gene expression data from the Allen Human Brain Atlas, we observed that regions with lower subcortical χ in schizophrenia had the highest expression of genes related to oligodendrocytes. As oligodendrocytes are the most iron-rich brain cells [61] and rely on iron for myelin production [62], low χ in schizophrenia may indicate oligodendrocyte loss or dysfunction and impaired myelin synthesis.

### Origin of lower subcortical χ in schizophrenia

Our finding of lower χ for the globus pallidus [14, 17], SN-VTA [13, 17, 27], putamen [14, 17], and, for the first time, the caudate nucleus in schizophrenia are consistent with previous iron-sensitive MRI studies in early-stage illness. These results contrast with a study showing increased putaminal χ in patients with chronic schizophrenia (>5 years duration) compared to controls [15]. As our sample was mostly recent-onset schizophrenia, this discrepancy may reflect the effects of chronic illness. However, we found no association between illness duration and subcortical χ in our sample. Another sample difference that may contribute to the discrepant findings is alcohol use, which is linked to elevated subcortical χ [63], given that patients reported higher Alcohol, Smoking, and Substance Involvement Screening Test scores than controls in the prior study. With the limited number of chronic patients (n = 14; 18%) in our study and the small sample size in the chronic study (controls n = 12; chronic schizophrenia n = 14), further research in larger cohorts is warranted to clarify these potential illness duration-related changes.

By showing that mean diffusivity was greater in the subcortex for the schizophrenia group, our results suggest that schizophrenia is associated with less subcortical paramagnetic material (i.e., iron) rather than more diamagnetic matter (i.e., myelin). This theory is supported by Sui et al. [17] who, using a multimodal MRI approach capable of uncoupling iron and myelin signal, linked a diagnosis of schizophrenia (n = 15) to lower basal ganglia iron in the absence of myelin differences when compared to matched controls (n = 35). By conducting the first δχ calculation in a schizophrenia cohort, we showed that white matter δχ was significantly lower compared to controls. This provides novel evidence that myelin levels are lower in schizophrenia. Other brain studies have linked schizophrenia to higher mean diffusivity [64], altered myelin-associated gene expression [65, 66], and decreased myelin postmortem staining [67, 68]. The convergence of these findings indicates that lower χ in early-stage illness is due to lower iron levels, which remain observable despite less myelin, which would otherwise increase χ.

As we discussed in our recent publication [27], schizophrenia is associated with higher NM-MRI signal (indexing neuromelanin-bound iron) and lower χ in the SN-VTA, suggesting lower non-neuromelanin-bound iron in the condition. Most brain tissue iron is bound to ferritin [61], with levels rising sharply from birth to adolescence and plateauing in the mid-20s [20, 69, 70]. Iron-sensitive MRI studies in adolescents and adults under 30 have shown correlations between brain iron levels and both cognitive function [69] and the maturation of the striatal dopamine system [70], both of which are frequently impaired in schizophrenia [1]. Therefore, our findings of lower subcortical χ in patients with schizophrenia, with a mean age of 32 years, may reflect a disruption in the normal developmental trajectory of brain iron accumulation.

Our gene expression analysis revealed that regions of lower χ in schizophrenia had higher expression of genes related to oligodendrocytes—the brain cells with the highest iron concentration [61]. Oligodendrocyte dysfunction in schizophrenia is supported by postmortem findings that lower oligodendrocyte concentrations in the anterior putamen [71], caudate nucleus [72], and prefrontal cortex [73–75], along with reduced expression of oligodendrocyte-related genes and proteins in various brain regions in schizophrenia relative to controls [62, 76, 77].

We interpret that low basal ganglia χ in schizophrenia reflects low iron in oligodendrocytes. Given the primary role of oligodendrocytes in myelin synthesis and maintenance via iron-dependent pathways [62], this likely disrupts myelin production, contributing to the lower white matter δχ observed in schizophrenia. Further, our GO analysis revealed the highest enrichment of glutamatergic synapse-related genes where χ was most reduced in schizophrenia. Since glutamate function, known to be altered in schizophrenia [78, 79], influences oligodendrocyte-mediated myelination and is regulated by oligodendrocyte-expressed glutamate transporters [80], low iron in oligodendrocytes may be linked to altered glutamate function.

### Origin of higher subcortical χ in schizophrenia

Although we did not find significantly higher χ for any ROI in schizophrenia, we identified clusters in the right anterior thalamus and right nucleus accumbens where this effect was present. This finding contrasts with studies reporting lower thalamic χ [13] and R2* in schizophrenia [17], though they only examined the region as a whole. Interestingly, a previous study using R2* with a voxelwise approach identified an increased iron-sensitive signal in the right anterior thalamus [16].

Postmortem analyses have shown that the thalamus is the most myelin-rich yet iron-poor [20, 21] subcortical region. This likely explains why χ has a strong negative correlation with myelin levels in the thalamus, which is not observed in the basal ganglia [21]. Our finding of an overall negative χ in the thalamus supports the notion that myelin contributes more substantially to χ than iron in this region, making χ here potentially more sensitive to myelin differences. Consequently, the cluster of increased χ detected in schizophrenia may primarily reflect myelin loss rather than iron accumulation. This interpretation is supported by the observation that thalamic mean diffusivity was higher in schizophrenia, although this increase did not remain significant after correction for multiple comparisons. Similarly, the nucleus accumbens—containing the other cluster where schizophrenia was associated with elevated χ—showed an overall negative χ. While iron and myelin concentrations have not been robustly quantified in the nucleus accumbens, a negative χ suggests that myelin changes may have a more significant influence on overall χ in this region.

The anterior thalamus, a key hub in the cortico-basal ganglia network, receives a bulk of the basal ganglia GABAergic output [81] and projects to the medial prefrontal cortex and other limbic structures [82–84]. Schizophrenia-related dysconnectivity in this network may result from an altered excitation-inhibition balance [85] driven by basal ganglia hyperdopaminergia [2], medial prefrontal cortical hypoglutamatergia [79], and thalamic hyperglutamergia [79]. Voxels with higher χ in schizophrenia were enriched for genes related to inhibitory neurons, excitatory neurons, microglia, and schizophrenia-related GO terms [60]. Given the association between cortico-basal ganglia dysconnectivity and impaired white matter integrity in schizophrenia [86], we propose that increased anterior thalamic χ in schizophrenia may reflect myelin loss resulting from an excitation-inhibition imbalance.

### Methodological considerations

Tissue χ values differ based on the QSM postprocessing method used [40], so comparing our numerical values with other studies should be done cautiously. We used a previously implemented pipeline [87, 88], validated to locate iron reductions in resected brain tissue [87], and incorporated the best practice postprocessing steps [40]. While myelin orientation affects χ, this is negligible in tissues with fractional anisotropy <0.6 [89], where higher values are unlikely outside of white matter tracts [90]. Therefore, group differences in subcortical χ are unlikely to be due to the orientation of fibers running through these structures.

Based on ex vivo findings that χ becomes more negative as white matter tracts align perpendicularly to the background magnetic field [22–24], δχ values should be positive. Consistent with prior in vivo studies, we observed positive δχ in the optic radiation and splenium of the corpus callosum [44, 45], but a negative δχ in the corticospinal tract [45] and a non-significant δχ in the anterior limb of the internal capsule [45]. These findings in the latter two regions are likely due to χ artifacts from the nearby paramagnetic basal ganglia [25]. Additionally, we identified significantly positive δχ in the superior longitudinal fasciculus and cingulum, which avoids these paramagnetic structures. Our use of iterative Tikhonov dipole inversion [91] for χ calculation rather than the Susceptibility Tensor Imaging (STI) toolbox [92] implemented in previous studies may explain why we detected positive δχ in these additional tracts. The absence of positive δχ in the genu and body of the corpus callosum could result from the non-linear orientation-dependence of χ in these tracts [23, 45]. Similarly, the lack of a significant δχ in the anterior thalamic radiation may be due to an absence of orientation-dependence for χ here [45].

Previous studies utilizing both QSM and R2* in schizophrenia have identified lower χ in the absence of R2* difference [13, 14]. Given that R2* correlates with both iron and myelin levels, while χ positively associates with iron and negatively with myelin [21, 93], concurrent iron loss and myelin gain could explain these results. In our study, we also observed lower χ in schizophrenia across several ROIs without significant R2* differences. However, given our findings suggesting low myelin in schizophrenia, a myelin increase is unlikely to account for this pattern. It is possible that χ is more sensitive to iron changes than R2*. This is made more likely by the demonstration that R2* but not χ is influenced by factors such as the microscopic distribution of iron [94], oxidation state of iron [95, 96], and local water concentration [97].

## Conclusion

Our study provides evidence that lower subcortical χ in schizophrenia is due to lower iron levels, despite lower myelin levels in this region. Our exploratory gene expression analysis linked gene variants affecting oligodendrocytes, iron-rich brain cells that synthesize myelin, to these findings. These findings point to oligodendrocyte dysfunction as a potential factor in disease pathophysiology.

## Supplementary information

Supplemental Materials

## Data Availability

The data and code that support the findings of this study are available upon reasonable request.

## Code availability

The data and code that support the findings of this study are available upon reasonable request.
