# Supplementary material for: The role of low subcortical iron, white matter myelin, and oligodendrocytes in schizophrenia: a quantitative susceptibility mapping and diffusion tensor imaging study
Source: Mol Psychiatry. 2025 Sep 5;31(2):941–52. doi: 10.1038/s41380-025-03195-7 (PMC12815684; doi:10.1038/s41380-025-03195-7)
Supplement: Supplementary file 1 — Supplemental Materials [file 41380_2025_3195_MOESM1_ESM.docx]

**Supplementary Figures**


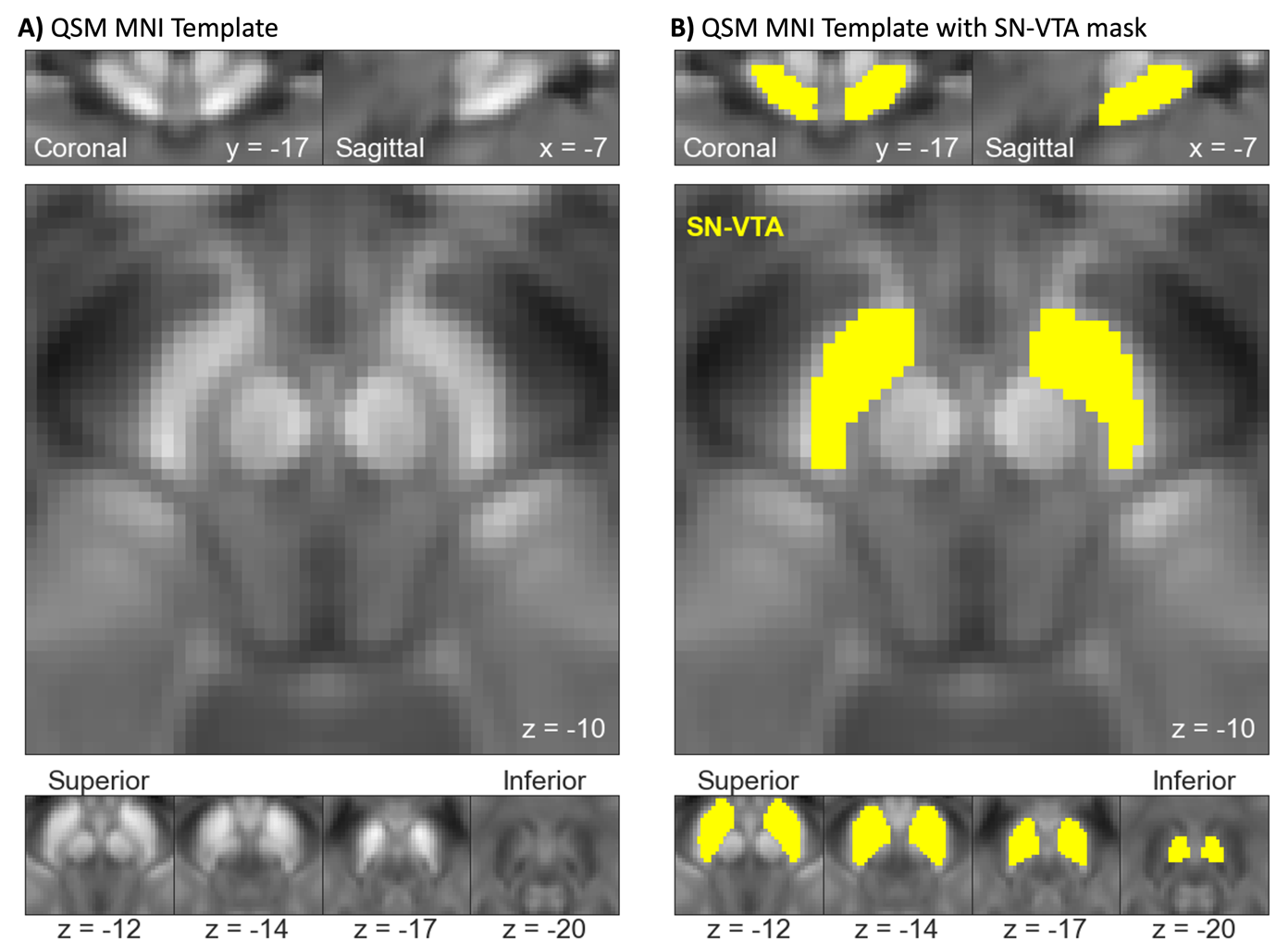


**Figure S1. A)** The study quantitative susceptibility mapping (QSM) template produced by averaging QSM images normalized to the Montreal Neurological Imaging (MNI) space. The template is displayed in the MNI space at the annotated orientation and slice level. **B)** The substantia nigra and ventral tegmental area (yellow; SN-VTA) mask is displayed on the template.

**Figure S2.** Fiber-to-field angle (θ) of voxels within the processed masks for white matter regions of interest for a single control. GCC, genu of the corpus callosum; BCC, body of the corpus callosum; SCC, splenium of the corpus callosum; ALIC, anterior limb of the internal capsule; ATR, anterior thalamic radiation; CST, corticospinal tract; SLF, superior longitudinal fascicle; CG, cingulum; OR, optic radiation; WM, all white matter tracts combined.

**Figure S3.** Box plots showing the magnetic susceptibility (χ) region of interest results. Data for healthy controls are in blue, and for the patients with schizophrenia they are in orange. ppb, parts per billion; SN-VTA, substantia nigra and ventral tegmental area; ns, non-significant (p>0.05); *, p<0.05; **, p<0.01; ***, p<0.001.

|  | **Striatal Subregion Magnetic Susceptibility (χ; ppb) Results** | | | | | | |
| --- | --- | --- | --- | --- | --- | --- | --- |
|  | **Controls** | | **Patients** | | **Test Statistics** | | |
|  | **Mean** | **SD** | **Mean** | **SD** | **Cohen’s d** | **t-test** | **p-value** |
| Limbic | 16.53 | 7.36 | 16.6 | 8.57 | -0.01 | 0.05 | 0.958 |
| Associative | 34.89 | 8 | 31.77 | 6.96 | -0.42 | -2.62 | 0.029 |
| Sensorimotor | 51.6 | 14.6 | 47.32 | 13.81 | -0.3 | -1.89 | 0.091 |

**Table S1.** Case-control analysis of χ for the striatal functional subregions. P-values corrected for multiple comparisons using the Benjamini-Hochberg method. ppb, parts per billion.

**Figure S4.** Box plots showing the mean diffusivity subcortical region of interest results. Data for healthy controls are in blue, and for the patients with schizophrenia they are in orange. SN-VTA, substantia nigra and ventral tegmental area; ns, non-significant (p>0.05); *, p<0.05; * (ns), significant prior to false discovery rate (FDR) correction for multiple comparisons.

**Figure S5:** Clusters where magnetic susceptibility (χ) was significantly different in schizophrenia relative to controls. These clusters were identified by threshold-free cluster enhancement (TFCE) combined with permutation testing (10,000 iterations) and significance determined with false discovery rate correction (p<0.05). Schizophrenia was associated with higher χ than controls for clusters in the right prefrontal thalamus (Rt THA) and right nucleus accumbens (Rt NA). For the eight other identified clusters, schizophrenia was associated with lower χ. Clusters are displayed on the T1-weighted Montreal Neurological Imaging (MNI) template at the annotated MNI coordinates. Lt, left; Rt, right; SN-VTA, substantia nigra and ventral tegmental area; LN, lenticular nucleus (putamen and globus pallidus combined); CN, caudate nucleus.

| **ROI** | **n voxels** | **t-value** | **x** | **y** | **z** |
| --- | --- | --- | --- | --- | --- |
| Lt LN | 1927 | -4.98 | -21 | -3 | 4 |
| Rt LN | 2386 | -4.64 | 20 | -3 | -4 |
| Lt SN-VTA 2 | 183 | -4.16 | -6 | -15 | -15 |
| Lt CN 1 | 323 | -3.86 | -11 | 22 | -3 |
| Rt SN-VTA | 234 | -3.56 | 7 | -22 | -20 |
| Lt CN 2 | 212 | -3.24 | -8 | 10 | 7 |
| Lt SN-VTA 1 | 70 | -2.82 | -11 | -23 | -13 |
| Rt NA | 48 | 3.73 | 23 | 5 | -11 |
| Rt THA | 108 | 3.92 | 5 | -8 | 4 |

**Table S2.** Threshold-free cluster enhancement (TFCE) identified clusters where schizophrenia was associated with different magnetic susceptibility (χ) than controls. Coordinates refer to the peak value in each cluster in the Montreal Neurological Imaging (MNI) space. ROI, regions of interest; n voxels, number of voxels in cluster; Lt, left; Rt, right; LN, lenticular nucleus (putamen and globus pallidus combined); SN-VTA, substantia nigra and ventral tegmental area; CN, caudate nucleus; NA, nucleus accumbens; THA, thalamus.

| Variable | Coefficient | Standard Error | t-score | p-value | [95% CI] | |  |
| --- | --- | --- | --- | --- | --- | --- | --- |
| (Intercept) | 20.79 | 2.82 | 7.38 | <0.001 | 15.27 | 26.31 |  |
| Current Smoker | 0.81 | 1.38 | 0.58 | 0.559 | -1.9 | 3.51 |  |
| Past Smoker | -0.16 | 1.84 | -0.09 | 0.93 | -3.77 | 3.45 |  |
| THC-positive UDS | -0.09 | 1.4 | -0.06 | 0.95 | -2.83 | 2.65 |  |
| Male Sex | 3.11 | 1.23 | 2.43 | 0.015 | 0.06 | 5.61 |  |
| Age | 0.13 | 0.09 | 1.49 | 0.135 | -0.04 | 0.3 |  |
| Duration of illness | 0.14 | 0.15 | -0.98 | 0.327 | -0.43 | 0.14 |  |

**Table S3.** Results from the robust linear model built to predict subcortical magnetic susceptibility (χ) in the patient population (schizophrenia n=79) with duration of illness and potential confounders.

**Figure S6.** Heatmap showing the Pearson’s r correlation between region of interest (ROI) magnetic susceptibility (χ), measured by quantitative susceptibility mapping (QSM), with clinical variables in the schizophrenia group. None were statistically significant when corrected for multiple comparisons using the Benjamini-Hochberg method. SN-VTA, substantia nigra and ventral tegmental area; PANSS, Positive and Negative Syndrome Scale; BNSS, Brief Negative Symptoms Scale.

|  | **Subcortical ROI R2* Results** | | | | | | |
| --- | --- | --- | --- | --- | --- | --- | --- |
|  | **Controls** | | **Patients** | | **Test Statistics** | | |
|  | **Mean** | **SD** | **Mean** | **SD** | **Cohen’s d** | **t-test** | **p-value** |
| Whole Subcortex | 22.9 | 1.52 | 22.51 | 1.28 | -0.28 | -1.78 | 0.077 |
| Thalamus | 20.6 | 1.14 | 20.49 | 1.12 | -0.1 | -0.61 | 0.541 |
| Caudate Nucleus | 20.55 | 1.78 | 20.14 | 1.65 | -0.24 | -1.48 | 0.140 |
| Putamen | 23.49 | 2.04 | 23.04 | 1.88 | -0.23 | -1.46 | 0.147 |
| Nucleus Accumbens | 17.49 | 2.11 | 17.34 | 2.02 | -0.07 | -0.44 | 0.659 |
| Globus Pallidus | 36.54 | 4.41 | 34.91 | 4 | -0.39 | -2.45 | 0.016 |
| SN-VTA | 30.08 | 3.51 | 29.21 | 3.46 | -0.25 | -1.57 | 0.118 |

**Table S4.** Comparison of mean tissue effective transverse relaxation rate (R2*) for subcortical regions of interest (ROI) between groups. P-values corrected for multiple comparisons using the Benjamini-Hochberg method. SD, standard deviation; SN-VTA, substantia nigra and ventral tegmental area.

|  | **Subcortical ROI Fractional Anisotropy Results** | | | | | | |
| --- | --- | --- | --- | --- | --- | --- | --- |
|  | **Controls** | | **Patients** | | **Test Statistics** | | |
|  | **Mean** | **SD** | **Mean** | **SD** | **Cohen’s d** | **t-test** | **p-value** |
| Whole Subcortex | 0.261 | 0.01 | 0.255 | 0.01 | -0.56 | -2.96 | 0.014 |
| Thalamus | 0.313 | 0.012 | 0.306 | 0.014 | -0.59 | -3.16 | 0.014 |
| Caudate Nucleus | 0.228 | 0.015 | 0.22 | 0.018 | -0.50 | -2.65 | 0.022 |
| Putamen | 0.188 | 0.011 | 0.186 | 0.01 | -0.14 | -0.72 | 0.475 |
| Nucleus Accumbens | 0.207 | 0.023 | 0.201 | 0.022 | -0.16 | -0.83 | 0.475 |
| Globus Pallidus | 0.317 | 0.017 | 0.311 | 0.019 | -0.32 | -1.70 | 0.129 |
| SN-VTA | 0.464 | 0.025 | 0.457 | 0.026 | -0.32 | -1.71 | 0.129 |

**Table S5.** Comparison of mean fractional anisotropy for subcortical regions of interest (ROI) between groups. P-values corrected for multiple comparisons using the Benjamini-Hochberg method. SD, standard deviation; SN-VTA, substantia nigra and ventral tegmental area.

**Figure S7.** Box plots showing the fractional anisotropy subcortical region of interest results. Data for healthy controls are in blue, and for the patients with schizophrenia they are in orange; SN-VTA, substantia nigra and ventral tegmental area; ns, non-significant (p>0.05); *, p<0.05

**Figure S8.** Histograms showing the group average distribution of fibre-to-field angles (θ; in degrees) for voxels within each white matter region of interest (ROI) for controls. GCC, genu of the corpus callosum; BCC, body of the corpus callosum; SCC, splenium of the corpus callosum; ALIC, anterior limb of the internal capsule; ATR, anterior thalamic radiation; CST, corticospinal tract; SLF, superior longitudinal fascicle; CG, cingulum; OR; optic radiation.

**Figure S9.** Orientation dependence of white matter magnetic susceptibility (χ). Line plots illustrating the relationship between χ and fiber-to-field angle (θ, in degrees) across selected white matter regions of interest. Values were generated from the 10 equally populated bins based on the quantiles of the θ distribution. The mean θ of each bin was plot on the x-axis and mean bin χ displayed on the y-axis. The bold colored line represents the average χ values for all voxels in the control group, with error bars indicating the standard deviation. Translucent lines depict individual plots for each control participant. As theoretically hypothesized, χ became more negative as θ increased in the SCC (R^2^= 0.55), SLF (R^2^= 0.84), CG (R^2^= 0.92), and OR (R^2^= 0.94). The opposite relationship was seen in the CST (R^2^=0.94), GCC (R^2^= 0.75), and ALIC (R^2^= 0.9). No relationship was identified in the BCC (R^2^= 0.03) or ATR (R^2^= 0.13). GCC, genu of the corpus callosum; BCC, body of the corpus callosum; SCC, splenium of the corpus callosum; ALIC, anterior limb of the internal capsule; ATR, anterior thalamic radiation; CST, corticospinal tract; SLF, superior longitudinal fascicle; CG, cingulum; OR; optic radiation; R^2^, r squared.

| **ROI** | **χ Mean** | **χ SE** | **χ [95% CI]** | | **χiso Coef** | **χiso SE** | **χiso [95% CI]** | | **Χiso p** | **δχ Coef** | **δχ SE** | **δχ [95% CI]** | | **δχ p** | **R²** |
| --- | --- | --- | --- | --- | --- | --- | --- | --- | --- | --- | --- | --- | --- | --- | --- |
| **OR** | -37.79 | 0.07 | -32.35 | -32.07 | -35.63 | 0.5 | -36.78 | -34.48 | <0.001 | 32.14 | 2.75 | 25.8 | 38.48 | <0.001 | 0.94 |
| **CG** | -23.52 | 0.05 | -18.99 | -18.77 | -28.19 | 1.27 | -31.11 | -25.27 | <0.001 | 26.36 | 2.73 | 20.07 | 32.64 | <0.001 | 0.92 |
| **SCC** | -29.84 | 0.08 | -31.02 | -30.7 | -31.55 | 0.36 | -32.38 | -30.72 | <0.001 | 13.35 | 4.26 | 3.52 | 23.17 | 0.014 | 0.55 |
| **SLF** | -14.77 | 0.04 | -11.54 | -11.38 | -16.78 | 0.95 | -18.97 | -14.59 | <0.001 | 9.93 | 1.53 | 6.41 | 13.45 | <0.001 | 0.84 |
| **ATR** | -14.69 | 0.09 | -14.99 | -14.65 | -15.54 | 0.85 | -17.51 | -13.57 | <0.001 | 2.57 | 2.37 | -2.89 | 8.02 | 0.31 | 0.13 |
| **BCC** | -32.69 | 0.11 | -40.3 | -39.88 | -39.29 | 2.27 | -44.52 | -34.05 | <0.001 | -4.85 | 10.6 | -29.3 | 19.59 | 0.659 | 0.03 |
| **CST** | -21.48 | 0.06 | -37.11 | -36.87 | -19.26 | 1.64 | -23.03 | -15.49 | <0.001 | -25.77 | 2.24 | -30.93 | -20.61 | <0.001 | 0.94 |
| **ALIC** | -34.8 | 0.1 | -44.88 | -44.5 | -29.87 | 2.18 | -34.9 | -24.84 | <0.001 | -36.96 | 4.31 | -46.91 | -27.01 | <0.001 | 0.9 |
| **GCC** | -9.14 | 0.08 | -17.99 | -17.66 | -13.51 | 1.37 | -16.67 | -10.36 | <0.001 | -54.78 | 11.25 | -80.73 | -28.82 | 0.001 | 0.75 |

**Table S6.** Results from the least squares regression model estimating magnetic susceptibility anisotropy (δχ) from for each white matter tract in the healthy controls. Magnetic susceptibility (χ) in each tract and estimated orientation-independent χ (χiso) is also reported. OR, optic radiation; CG, cingulum; SCC, splenium of the corpus callosum; SLF, superior longitudinal fascicle; ATR, anterior thalamic radiation; BCC, body of the corpus callosum; CST, corticospinal tract; ALIC, anterior limb of the internal capsule; GCC, genu of the corpus callosum; p, p-value; R^2^, r squared.

|  | **χ Mean** | **χ SE** | **χ [95% CI]** | | **χiso Mean** | **χiso SE** | **χiso [95% CI]** | | **δχ Mean** | **δχ SE** | **δχ [95% CI]** | |
| --- | --- | --- | --- | --- | --- | --- | --- | --- | --- | --- | --- | --- |
| Controls | -25.75 | 0.69 | -27.13 | -24.36 | -27.71 | 0.6 | -28.92 | -26.51 | 19.06 | 2.1 | 14.82 | 23.3 |
| Patients | -24.8 | 0.49 | -25.78 | -23.82 | -27.12 | 0.41 | -27.94 | -26.31 | 13.29 | 1.96 | 9.39 | 17.19 |
| d | -0.22 |  |  |  | -0.16 |  |  |  | -0.37 |  |  |  |
| t-value | -1.122 |  |  |  | -0.812 |  |  |  | -2.012 |  |  |  |
| p-value | 0.419 |  |  |  | 0.419 |  |  |  | 0.047 |  |  |  |

**Table S7.** Analysis of the white matter tracts in control participants and those with schizophrenia. Magnetic susceptibility (χ), orientation-independent χ (χiso), and magnetic susceptibility anisotropy (δχ) were initially calculated for each of the specified tracts (splenium of the corpus callosum, superior longitudinal fasciculus, cingulate, and optic radiation) for each participant. These values were then averaged across tracts to generate individual participant values for χ, χiso, and δχ. The case-control comparisons and associated statistics presented here were derived from these participant-level averages. P-values for group differences in χ and χiso were adjusted for multiple comparisons using the Benjamini-Hochberg method, in consideration of the exploratory nature of these analyses. d, Cohen’s d effect size.

|  | **White Matter Diffusion Tensor Imaging Results** | | | | | | |
| --- | --- | --- | --- | --- | --- | --- | --- |
|  | **Controls** | | **Patients** | | **Test Statistics** | | |
|  | **Mean** | **SD** | **Mean** | **SD** | **Cohen’s d** | **t-test** | **p-value** |
| Skeletal White Matter Fractional Anisotropy | 0.417 | 0.012 | 0.411 | 0.014 | -0.45 | -2.41 | 0.036 |
| Skeletal White Matter Mean Diffusivity | 77.55 | 1.71 | 77.87 | 1.62 | 0.19 | 0.99 | 0.325 |

**Table S8.** Group white matter fractional anisotropy and mean diffusivity results. P-values corrected for multiple comparisons using the Benjamini-Hochberg method. SD, standard deviation.

**Figure S10.** Scatter plot showing the positive correlation between subcortical magnetic susceptibility (χ) and age across all participants in the study (r=0.21, p=0.008).

| **Gene set name** | **Domain** | **N (genes)** | **P (Bonferroni-corrected)** |
| --- | --- | --- | --- |
| GO:0034702_ion_channel_complex | Cellular Component | 229 | 1.01E-05 |
| GO:0045202_synapse | Cellular Component | 577 | 5.46E-05 |
| GO:0034703_cation_channel_complex | Cellular Component | 199 | 5.77E-05 |
| GO:1902495_transmembrane_transporter_complex | Cellular Component | 248 | 7.75E-05 |
| GO:1990351_transporter_complex | Cellular Component | 256 | 2.58E-04 |
| GO:0030424_axon | Cellular Component | 308 | 3.87E-04 |
| GO:0022839_ion_gated_channel_activity | Molecular Function | 245 | 5.70E-04 |
| GO:0022843_voltage-gated_cation_channel_activity | Molecular Function | 118 | 7.40E-04 |
| GO:0043005_neuron_projection | Cellular Component | 751 | 8.52E-04 |
| GO:0022832_voltage-gated_channel_activity | Molecular Function | 131 | 9.64E-04 |
| GO:0005244_voltage-gated_ion_channel_activity | Molecular Function | 131 | 9.64E-04 |
| GO:0036477_somatodendritic_compartment | Cellular Component | 403 | 1.35E-03 |
| GO:0045664_regulation_of_neuron_differentiation | Biological Process | 378 | 3.87E-03 |
| GO:0030425_dendrite | Cellular Component | 308 | 4.43E-03 |
| GO:0097447_dendritic_tree | Cellular Component | 311 | 4.51E-03 |
| GO:0022836_gated_channel_activity | Molecular Function | 253 | 4.96E-03 |
| GO:0060341_regulation_of_cellular_localization | Biological Process | 620 | 0.012 |
| GO:0050804_modulation_of_chemical_synaptic_transmission | Biological Process | 233 | 0.013 |
| GO:0005245_voltage-gated_calcium_channel_activity | Molecular Function | 35 | 0.015 |
| GO:0098794_postsynapse | Cellular Component | 260 | 0.018 |
| GO:2001257_regulation_of_cation_channel_activity | Biological Process | 135 | 0.018 |
| GO:0007399_nervous_system_development | Biological Process | 1430 | 0.020 |
| GO:0099177_regulation_of_trans-synaptic_signaling | Biological Process | 234 | 0.022 |
| GO:0043025_neuronal_cell_body | Cellular Component | 184 | 0.023 |

**Table S9.** Gene ontology (GO) terms associated with schizophrenia risk generated from the Trubetskoy et al. genome-wide association study (GWAS) [1]

**Supplementary Material and Methods**

Image acquisition

We used the following MRI protocols: magnetization prepared rapid gradient echo (MPRAGE) for T1-weighted images (acquisition time (TA)=5 mins 35 secs, repetition time (TR)=2300ms, echo time (TE)=2.91ms, flip angle=9°, 176 slices with the voxel size=1x1x1mm^3^); a 3D gradient recall echo (GRE) sequence for the R2* and QSM maps (TA=8 mins 5 secs, TR=50ms, first TE at 5.84ms with 8 subsequent echoes 4.79ms apart, flip angle=15°, 144 slices with the voxel size=1x1x1mm^3^); and a single-shot echo-planar imaging DTI sequence (TA=7 mins 36 secs, TR=3200ms, TE=69ms, 27 slices with a voxel size=1.7x1.7x4mm^3^. 11 b=0 s/mm^2^ images were acquired and diffusion gradients were applied in 64 uniformly distributed directions with b=1000 s/mm^2^. A second acquisition was performed with reversed phase encoding direction, no diffusion gradients and otherwise identical parameters for distortion correction during pre-processing).

Processing of Quantitative Susceptibility Maps (QSM)

The initial echo time magnitude image from the 3D gradient recall echo was used to generate a brain mask with the FMRIB Software Laboratory (FSL) Brain Extraction Tool (BET) [2]. The phase images from all echo times were utilized to compute the frequency shift for each voxel by employing the Fit_ppm_complex.m function within the Morphology Enabled Dipole Inversion (MEDI) toolbox [3]. These frequency shifts, obtained from voxels within an eroded brain mask (approximately 95% of the size of the BET brain mask), were used to determine the local frequency shift via the projection onto dipole fields method [4]. The iterative Tikhonov dipole inversion method generated QSM maps from these local frequency shifts [5]. Given that 3D gradient recall echo sequences distort near significant B0 inhomogeneities, such as air-tissue or bone-tissue boundaries, the NiftyReg toolbox was employed for non-linear realignment of the magnitude brain to the T1-weighted brain, utilizing a low-resolution 15mm spline grid, targeting specifically the correction of distortions stemming from large B0 inhomogeneities [6].

Diffusion Tensor Imaging (DTI) Data Processing

DTI data was preprocessed using FSL’s diffusion toolbox [7]. Images were first corrected for effects from eddy current, distortion, and head movement [8]. Brain extraction was then completed before a diffusion tensor model was fit using dtifit and FSL’s tract-based spatial statistics (TBSS) toolbox [9] was then implemented to non-linear co-register of each subject’s fractional anisotropy (FA) image to the standard FMRIB58_FA_1mm template. An average fractional anisotropy white matter skeleton was created from these normalized images, using FSL’s recommended threshold of fractional anisotropy ≥0.2, which was transformed back into each participant’s subject space to later generate fractional anisotropy results. As the FMRIB58_FA_1mm template is in the MNI152 sixth-gen space the MNI152NLin2009cSym brain was non-linearly co-registered to the MNI152 sixth-gen T1-weighted template, and the subcortical atlas was moved along the relevant affine transformation-matrices and warp-fields until transformed to the subject’s DTI space for mean diffusivity calculations.

Our mask generation was in keeping with the methods used by Sibgatulin et al. [10, 11]. To generate the corpus callosum subregion masks, the commissural fiber probability map generated by TractSeg [12] for each participant was thresholded at a 90% probability level. A lateral ventricle mask, segmented by Freesurfer [13] (using labels 4 and 43), was then applied and expanded using a 6-voxel structural element. Next, the mask of the central sagittal plain of the corpus callosum was extracted from the Freesurfer segmentation (labels 251 to 255) with the dilated lateral ventricle mask removed to minimize segmentation error effects; this mask was further refined through opening with a 6-voxel element. The final corpus callosum mask was defined by morphologically dilating the sagittal plane mask with an ellipsoid of semiaxes 11 mm in the left-right and anterior-posterior directions, and 7 mm in the inferior-superior direction. The intersection of the sagittal plane and the commissural fiber mask was refined with a 6-voxel opening, after which the largest contiguous component was selected. The CC was then subdivided into the genu, body, and splenium, expanding the Freesurfer labels 255, 252-254, and 251, respectively, along the LR axis.

Additionally, the anterior limb of the internal capsule was isolated from the remaining anterior thalamic radiation using the participant’s Freesurfer subcortical gray matter segmentation mask, further expanded with a spherical element of an 8 mm radius.

Power calculation

Our power calculation indicated that a sample size of 78 participants per group would provide 80% power to detect a moderate effect size of 0.4 [14], with a significance level (α) set at 0.05. Consequently, we aimed to achieve this sample size for both cohorts undergoing QSM.

Red nucleus analysis

Given that prior studies have examined magnetic susceptibility (χ) and effective transverse relaxation rate (R2*) in the iron-rich red nucleus, we completed a case-control analysis using the Multi-modal-fused magnetic Susceptibility (MuSus-100) atlas to define the red nucleus [15]. Red nucleus χ was lower in patients with schizophrenia (mean=61.90, SD=26.30) than controls (mean=69.25, SD=24.84), showing a trend toward significance (d=-0.29, t=-1.81, p=0.072). There was no significant difference in red nucleus R2* between patients with schizophrenia (mean=27.34, SD=3.42) and controls (mean=27.97, SD=3.17; d=-0.19, t=-1.21, p=0.228).

**References**

1. Trubetskoy V, Pardiñas AF, Qi T, Panagiotaropoulou G, Awasthi S, Bigdeli TB, et al. Mapping genomic loci implicates genes and synaptic biology in schizophrenia. Nature. 2022;604:502–508.

2. Smith SM. Fast robust automated brain extraction. Hum Brain Mapp. 2002;17:143–155.

3. Liu T, Wisnieff C, Lou M, Chen W, Spincemaille P, Wang Y. Nonlinear formulation of the magnetic field to source relationship for robust quantitative susceptibility mapping. Magnetic Resonance in Medicine. 2013;69:467–476.

4. Liu T, Khalidov I, de Rochefort L, Spincemaille P, Liu J, Tsiouris AJ, et al. A novel background field removal method for MRI using projection onto dipole fields (PDF). NMR Biomed. 2011;24:1129–1136.

5. Karsa A, Punwani S, Shmueli K. An optimized and highly repeatable MRI acquisition and processing pipeline for quantitative susceptibility mapping in the head-and-neck region. Magnetic Resonance in Medicine. 2020;84:3206–3222.

6. Modat M, Cash DM, Daga P, Winston GP, Duncan JS, Ourselin S. Global image registration using a symmetric block-matching approach. J Med Imaging (Bellingham). 2014;1:024003.

7. Jenkinson M, Beckmann CF, Behrens TEJ, Woolrich MW, Smith SM. FSL. NeuroImage. 2012;62:782–790.

8. Andersson JLR, Sotiropoulos SN. An integrated approach to correction for off-resonance effects and subject movement in diffusion MR imaging. Neuroimage. 2016;125:1063–1078.

9. Smith SM, Jenkinson M, Johansen-Berg H, Rueckert D, Nichols TE, Mackay CE, et al. Tract-based spatial statistics: Voxelwise analysis of multi-subject diffusion data. NeuroImage. 2006;31:1487–1505.

10. Sibgatulin R, Güllmar D, Deistung A, Enzinger C, Ropele S, Reichenbach JR. Magnetic susceptibility anisotropy in normal appearing white matter in multiple sclerosis from single-orientation acquisition. NeuroImage: Clinical. 2022;35:103059.

11. Sibgatulin R, Güllmar D, Deistung A, Ropele S, Reichenbach JR. *In vivo* assessment of anisotropy of apparent magnetic susceptibility in white matter from a single orientation acquisition. NeuroImage. 2021;241:118442.

12. Wasserthal J, Neher P, Maier-Hein KH. TractSeg - Fast and accurate white matter tract segmentation. NeuroImage. 2018;183:239–253.

13. Desikan RS, Ségonne F, Fischl B, Quinn BT, Dickerson BC, Blacker D, et al. An automated labeling system for subdividing the human cerebral cortex on MRI scans into gyral based regions of interest. Neuroimage. 2006;31:968–980.

14. Brydges CR. Effect Size Guidelines, Sample Size Calculations, and Statistical Power in Gerontology. Innovation in Aging. 2019;3:igz036.

15. He C, Guan X, Zhang W, Li J, Liu C, Wei H, et al. Quantitative susceptibility atlas construction in Montreal Neurological Institute space: towards histological-consistent iron-rich deep brain nucleus subregion identification. Brain Struct Funct. 2022. 29 August 2022. https://doi.org/10.1007/s00429-022-02547-1.
